# Monocytes Phenotype and Cytokine Production in Human Immunodeficiency Virus-1 Infected Patients Receiving a Modified Vaccinia Ankara-Based HIV-1 Vaccine: Relationship to CD300 Molecules Expression

**DOI:** 10.3389/fimmu.2017.00836

**Published:** 2017-07-21

**Authors:** Joana Vitallé, Olatz Zenarruzabeitia, Iñigo Terrén, Montserrat Plana, Alberto C. Guardo, Lorna Leal, José Peña, Felipe García, Francisco Borrego

**Affiliations:** ^1^Immunopathology Group, BioCruces Health Research Institute, Barakaldo, Spain; ^2^AIDS Research Group, IDIBAPS, HIVACAT, Hospital Clìnic, University of Barcelona, Barcelona, Spain; ^3^Instituto Maimónides de Investigación Biomédica de Córdoba (IMIBIC), Córdoba, Spain; ^4^Ikerbasque, Basque Foundation for Science, Bilbao, Spain; ^5^Basque Center for Transfusion and Human Tissues, Galdakao, Spain

**Keywords:** human immunodeficiency virus, monocytes, CD300, CD300c, CD300f, therapeutic vaccine, lipopolysaccharide, HIV-1 vaccine

## Abstract

A modified vaccinia Ankara-based HIV-1 vaccine clade B (MVA-B) has been tested for safety and immunogenicity in low-risk human immunodeficiency virus (HIV)-uninfected individuals and as a therapeutic vaccine in HIV-1-infected individuals on combined antiretroviral therapy (cART). As a therapeutic vaccine, MVA-B was safe and broadly immunogenic; however, patients still showed a viral rebound upon treatment interruption. Monocytes are an important part of the viral reservoir and several studies suggest that they are partly responsible for the chronic inflammation observed in cART-treated HIV-infected people. The CD300 family of receptors has an important role in several diseases, including viral infections. Monocytes express CD300a, c, e, and f molecules and lipopolysaccharide (LPS) and other stimuli regulate their expression. However, the expression and function of CD300 receptors on monocytes in HIV infection is still unknown. In this work, we investigated for the first time the expression of CD300 molecules and the cytokine production in response to LPS on monocytes from HIV-1-infected patients before and after vaccination with MVA-B. Our results showed that CD300 receptors expression on monocytes from HIV-1-infected patients correlates with markers of HIV infection progression and immune inflammation. Specifically, we observed a positive correlation between the expression of CD300e and CD300f receptors on monocytes with the number of CD4+ T cells of HIV-1-infected patients before vaccination. We also saw a positive correlation between the expression of the inhibitory receptor CD300f and the expression of CD163 on monocytes from HIV-1-infected individuals before and after vaccination. In addition, monocytes exhibited a higher cytokine production in response to LPS after vaccination, almost at the same levels of monocytes from healthy donors. Furthermore, we also described a correlation in the expression of CD300e and CD300f receptors with TNF-α production in response to LPS, only in monocytes of HIV-1-infected patients before vaccination. Altogether, our results describe the impact of HIV-1 and of the MVA-B vaccine in cytokine production and monocytes phenotype.

## Introduction

The development of combined antiretroviral therapy (cART) has significantly improved the clinical outcome in human immunodeficiency virus (HIV)-infected patients. However, long-term cART poses considerable side effects and costs, and stopping the treatment generally causes rapid viral rebounds, mostly due to the latent viral reservoirs (1, 2). For this reason, several strategies are being studied in order to achieve a permanent control of HIV replication inducing an effective antiviral T cell response. Among the most immunogenic approaches for inducing HIV-specific CD8+ T cell responses have been poxvirus vector boost vaccines (3, 4). Recently, a modified vaccinia Ankara vector expressing HIV-1 antigens clade B (MVA-B) was tested as a therapeutic vaccine. MVA-B was first tested with healthy volunteers (RISVAC02), which demonstrated that this vaccine was safe, well tolerated (5) and induced polyfunctional and durable T cell responses in most individuals (6). Importantly, it has also been tested as a therapeutic vaccine in a phase-I clinical trial in HIV-1-infected individuals on cART (RISVAC03), and the vaccination with MVA-B vaccine was also safe and broadly immunogenic. Nevertheless, HIV-1-infected patients still showed a viral rebound upon treatment interruption, and vaccination did not affect the viral reservoir even in combination with disulfiram, a drug able to reactivate latent HIV-1 (7, 8). The viral rebound after removal of cART has been linked to the fact that vaccination with MVA-B tips the balance between activation and regulation toward regulation of the response of HIV-specific CD8+ T cells (9). Nevertheless, in order to design more effective therapeutic vaccines, more studies are required to completely understand the effects on the host of the MVA-B vaccination.

Although latently infected CD4+ T cells comprise the majority of the HIV reservoir, monocytes (mainly CD16+ monocytes) provide an important part of this reservoir and also perpetuate HIV replication through ongoing cell-to-cell transfer of virions and efficient infection of CD4+ T cells, even in the presence of cART (10). In addition, recent studies suggest that monocytes are also responsible for the chronic inflammation in cART-treated HIV-infected people (11). In fact, it has been described that monocytes of chronically HIV-infected subjects differ from monocytes of healthy people in subsets distribution (12), expression of different markers (e.g., CD163) (13), and cytokine production (e.g., IL-6) (11). All these findings emphasize the importance of studying the mechanisms that regulate the activation of monocytes in HIV-infected patients.

The human CD300 molecules (a, b, c, d, e, f, g, h) are type I transmembrane proteins that, with the exception of CD300g which is expressed on endothelial cells, are found in both lymphoid and myeloid cell lineages. CD300a and CD300f are inhibitory receptors while CD300b, CD300c, CD300d, CD300e, and CD300h are activating receptors (14–16). Inhibitory receptors contain a long cytoplasmic tail with immunoreceptor tyrosine-based inhibitory motifs (ITIMs) which are required for the inhibitory signaling. Activating receptors have a short cytoplasmic tail with a charged transmembrane amino acidic residue, that allows their association with adaptor proteins containing immunoreceptor tyrosine-based activating motifs and other activating motifs which induce activation signals (14, 16). CD300 molecules have an important role in several diseases, including viral infections (14, 16, 17). In the context of HIV infection, there are few publications describing the role of CD300 family. In HIV-infected patients, the expression of the CD300a inhibitory receptor is down-regulated on B lymphocytes, which may help to explain the hyperactivation and dysfunction of B cells observed in these individuals (18). Another important detail about CD300a involvement in the pathogenesis of HIV infection is given by the description of a positive correlation between mRNA levels of CD300a and the expression of BATF, a transcription factor that inhibit T cell function, in HIV-specific CD8+ T cells (19).

At least, monocytes express four members of this family: the CD300a and CD300f inhibitory receptors, and the CD300c and CD300e activating receptors. Among others, age and lipopolysaccharide (LPS) regulate the expression of these receptors (14, 16, 20). However, in HIV infection, the expression and function of CD300 receptors on monocytes is still unknown. In this work, we have analyzed the expression of CD300 molecules on monocytes from chronically HIV-1-infected patients and calculated the correlation with markers of HIV-1 infection progression (CD4+ T cell count) and immune inflammation (CD163 expression). Moreover, we investigated the effect of the vaccination with MVA-B in the cytokine production of monocytes stimulated with LPS in HIV-infected subjects and we studied the correlation with the CD300 family of molecules expression. Our results may contribute to a better knowledge of monocytes dysfunction in HIV-1 infection and the influence of the MVA-B therapeutic vaccine in these cells.

## Patients and Methods

### Patients and Samples

Samples were obtained from HIV-1-infected patients enrolled in the RISVAC03 clinical trial (NCT01571466) (8). RISVAC03 is a double-blinded randomized phase-I trial in which cART-treated HIV-1-infected individuals received four intramuscular injections of MVA-B vaccine at weeks 0, 4, 16, and 36, combined with disulfiram for 3 months after the last dose of the vaccine. Specifically, in this study we have analyzed available frozen peripheral blood mononuclear cells (PBMCs) from eight HIV-1-infected patients before (week 0) and after last vaccination (week 48). Clinical data of HIV-1-infected patients are shown in Table 1. Frozen PBMCs from seven healthy donors (HD) available from the phase-I trial RISVAC02 (NCT00679497) (5) were also studied. Only cells from non-vaccinated healthy individuals were analyzed. The means of the percentages of viable cells after thawing were: 69.4 ± 4.55% (HD), 70.0 ± 3.33% (HIV-infected patients before vaccination), and 67.3 ± 3.59% (HIV-infected patients after vaccination). This study was approved by the Research Ethics Committee of Hospital Clìnic, Barcelona, Hospital Germans Trias i Pujol, Badalona and Hospital Gregorio Marañón, Madrid, Spain. All subjects that participated in RISVAC02 and RISVAC03 clinical trials provided written and signed informed consent (5, 8).

**Table 1:** Clinical data of HIV-1-infected patients.

Patient	Undetectable VL (years)	CD4+ T cells Nadir (cells/mm^3^)	CD4+ T cells before ART (cells/mm^3^)	CD4+ T cells baseline (cells/mm^3^)	Age	Sex	Weight (kg)	Coinfection hepatitis C virus	Time of known HIV infection (years)
101	9	179	368	541	49	M	74	No	14
103	1	290	489	530	50	M	69	No	10
107	2	274	274	866	41	M	68	No	12
108	2	396	396	823	33	M	73	No	12
109	4	645	688	1,179	39	M	65	No	6
110	12	376	376	1,238	40	F	56	No	15
111	3	296	396	632	44	M	78	No	6
112	2	507	680	794	39	M	60	No	3

*VL, viral load; ART, antiretroviral therapy; HIV, human immunodeficiency virus*.

### Flow Cytometry Analysis

The following anti-human fluorochrome conjugated antibodies were used for flow cytometric analysis: PE-Cy7 mouse anti-CD14 (clone MφP9), PerCP-Cy5.5 mouse anti-HLA-DR (clone G46-6), PE mouse anti-IL-1α (clone 364-3B3-14), and FITC rat anti-IL-6 (clone MQ2-13A5) from BD Biosciences; FITC mouse anti-CD16 (clone B73.1), BV421 mouse anti-CD163 (clone GHI/61), and APC mouse anti-TNFα (clone Mab11) from Biolegend; PE mouse anti-CD300a (clone E59.126) from Beckman Coulter; eFluor660 mouse anti-CD300c (clone TX45) from eBioscience; and APC mouse anti-CD300e (clone UP-H2) and PE mouse anti-CD300f (clone UP-D2) from Miltenyi Biotec. To test the viability of the cells, the 633–635 nm excitation LIVE/DEAD Fixable Near-IR Dead Cell Stain Kit (Life Technologies) was used. Frozen PBMCs from HD and HIV-1-patients were thawed, washed, and incubated at 37°C for 1–2 h in R10 (10% FBS and 1% Penicillin/Streptavidin in RPMI-1640 medium) medium with 10U of DNase (Sigma-Aldrich), in a concentration of 2 × 10^6^ cells/ml. Afterward, cells were stained first with the LIVE/DEAD kit in order to detect dead cells, and then, they were incubated with different fluorochrome conjugated antibodies. Both steps were carried out for 30 min on ice protected from the light. PBMCs were fixed with 4% of paraformaldehyde (Sigma-Aldrich) for 15 min at 4°C and washed two times with PBS. A FACSCanto II flow cytometer (BD Biosciences) was used for sample acquisition and data was analyzed with FlowJo 10.0.7 software (TreeStar).

### LPS Stimulation and Intracellular Cytokine Staining (ICS)

Peripheral blood mononuclear cells from HD and HIV-1-infected patients were cultured (10^6^ cells/ml) in R10 medium with 1 ng/ml of LPS (Sigma) for 5 h at 37°C, in the presence of GolgiStop protein transport inhibitor containing monensin, following manufacturer’s indications (BD Biosciences). After the stimulation, PBMCs were stained with LIVE/DEAD kit, followed by incubation with different fluorochrome conjugated antibodies for extracellular staining. In order to accomplish the ICS, cells were first permeabilized with Cytofix/Cytoperm Plus Kit following the manufacturer’s protocol (BD Biosciences) and then they were incubated with different fluorochrome conjugated antibodies for the detection of cytokines. Sample acquisition and data analysis were carried out as described before.

### Data Representation and Statistical Analysis

GraphPad Prism software (version 6.01) was used for graphical representation and statistical analysis. Data were represented in dot plot graphs and bar graphs showing the mean with SEM, and pie chart graphs. Values obtained from different subject groups were compared with non-parametric tests; the comparison between HD and HIV-1-infected patients’ data was made with the unpaired Mann–Whitney test; and differences between HIV-1-infected patients before and after vaccination were evaluated with the Wilcoxon matched-pairs signed rank test. Correlation analyses were done using the same software. In the case of cytokine production data, percentages of polyfunctional, mono-functional, and non-functional cells were obtained by a Boolean gate analysis with FlowJo software and the representation of these data were done using GraphPad Prism software.

## Results

### CD300 Receptors Expression on Monocytes from HIV-1-Infected Patients Correlates with Markers of HIV Infection Progression and Immune Inflammation

**Figure 1:**
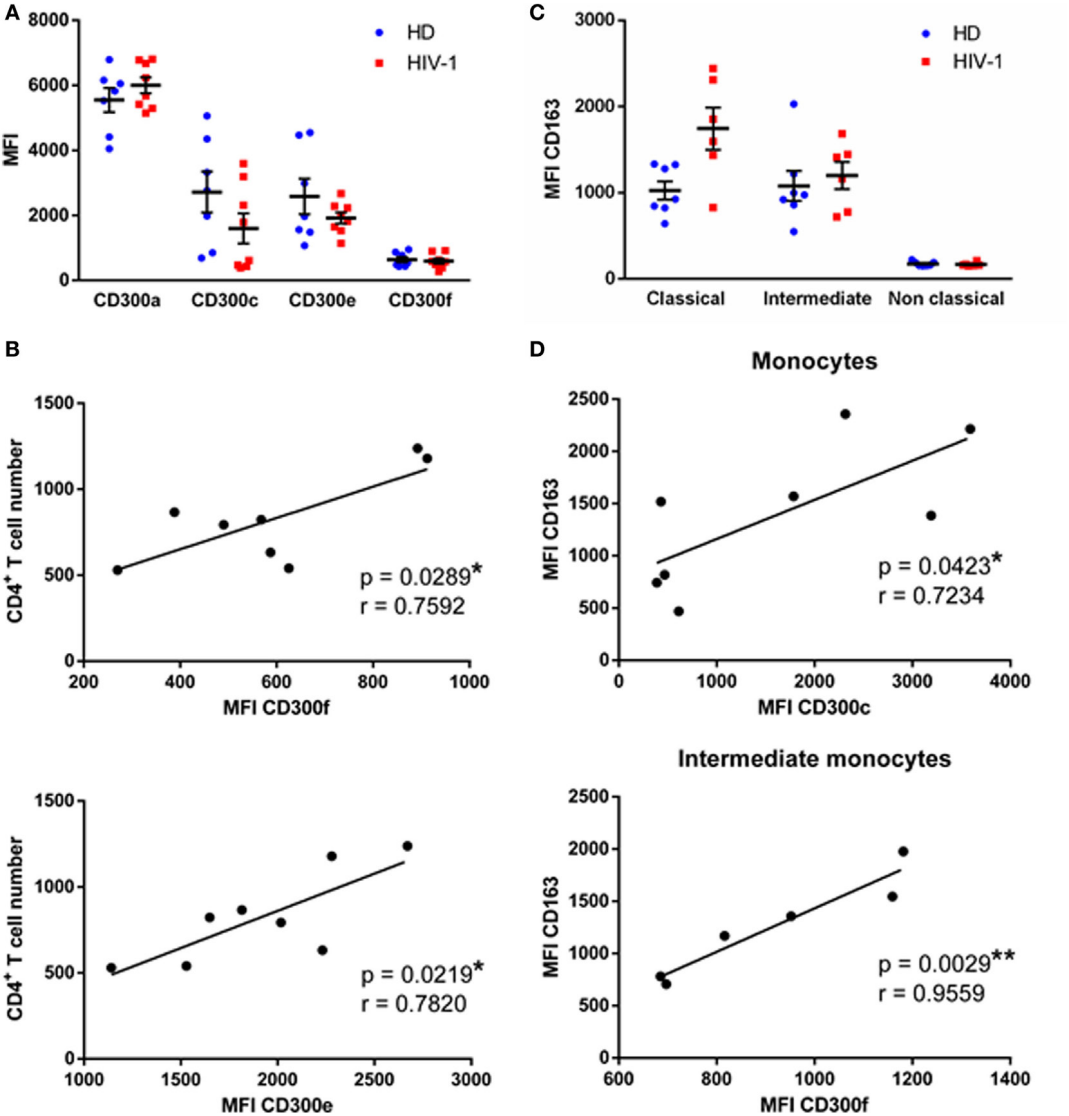
CD300 receptors expression in human immunodeficiency virus (HIV)-1-infected patients. **(A)** Dot plot graph presenting the median fluorescence intensity (MFI) of CD300a, CD300c, CD300e, and CD300f receptors expression on monocytes from healthy donors (HD) and HIV-1-infected patients. Each dot corresponds to an individual and the mean with the standard error of the mean (SEM) is shown. **(B)** Correlation between CD4+ T cell number at baseline of the study and the MFI of CD300f and CD300e receptors expression on monocytes from HIV-1-infected patients is represented; the linear regression is shown. **(C)** Dot plot graph representing the MFI of CD163 receptor expression on classical, intermediate, and non-classical monocytes from HD and HIV-1-infected individuals. Each dot corresponds to an individual and the mean with SEM is shown. **(D)** Correlation between the MFI of CD163 and CD300c receptors expression on total monocytes and CD163 and CD300f receptors expression on intermediate monocytes from HIV-1-infected patients; the linear regression is shown. *p < 0.05, **p < 0.01.

We first determined the expression of CD300a, CD300c, CD300e, and CD300f molecules on monocytes from HD and chronically HIV-1-infected subjects that are receiving cART at baseline, i.e., just before starting the RISVAC03 clinical trial. Monocytes were electronically gated based on their forward and side scatter properties, and the expression of CD14 and CD16; concretely, classical (CD14++ CD16−), intermediate (CD14++ CD16+), and non-classical (CD14+ CD16++) monocytes were analyzed (Figure S1A in Supplementary Material). As it has been described before (10, 12), the percentages of intermediate and non-classical monocytes were slightly increased in HIV-1-infected patients in comparison with HD (Figure S2 in Supplementary Material). The expression of four members of the CD300 receptor family was tested: the inhibitory receptors CD300a and CD300f, and the activating receptors CD300e and CD300c. We did not observed significant differences in the expression of CD300 receptors on monocytes of HIV-1-infected patients compared with HD (Figure 1A), not even when we separately analyzed each monocyte subpopulation (Figure S3 in Supplementary Material). In spite of that, we observed a tendency, although not statistically significant, of CD300c expression to decrease on monocytes of HIV-1-infected subjects [HD median fluorescence intensity (MFI) = 2,717 ± 630.4 vs HIV MFI = 1,596 ± 465.5] (Figure 1A), especially in non-classical monocytes (data not shown).

Next, we investigated the association between CD300 receptors expression and patients’ clinical features. Clinical data, which consists mainly of CD4+ T cell numbers, are shown in Table 1. CD300a and CD300c receptor expression on monocytes did not correlate with the number of CD4+ T cells at baseline (data not shown); however, the expression of CD300e (*p* < 0.05, *r* = 0.7820) and CD300f (*p* < 0.05, *r* = 0.7592) receptors was positively correlated with the CD4+ T cell numbers (Figure 1B).

Afterward, the expression of the CD163 receptor was analyzed and calculated the correlation with CD300 molecules expression in monocyte subpopulations. CD163 is a scavenger receptor, expressed exclusively on monocytes and macrophages, that has been investigated as a potential inflammation marker in different infectious diseases (13). In fact, sCD163 plasma levels are elevated in chronically HIV-1-infected patients and this has been related to a higher risk of comorbid disorders (11). We saw that CD163 expression of classical (HD MFI = 1,025 ± 106.7 vs HIV MFI = 1,744 ± 243.8) and intermediate (HD MFI = 1,079 ± 175.3 vs HIV MFI = 1,200 ± 158.2) monocytes was higher in HIV-1-infected subjects than in HD; unlike non-classical monocytes, which exhibited a very low expression in both groups (Figure 1C). Correlation analysis showed that in monocytes of HD, CD163 and CD300 receptors expression were not associated (data not shown). In contrast, there was a positive correlation between CD163 and CD300c expression (*p* < 0.05, *r* = 0.7234) on monocytes of HIV-1-infected subjects, and also between CD163 and CD300f expression (*p* < 0.01, *r* = 0.9559) in intermediate monocytes of HIV-1-patients (Figure 1D).

### Effects of MVA-B Vaccination on Monocytes from HIV-Infected Subjects

The safety and immunogenicity of the MVA-B vaccine in chronically HIV-1-infected patients and healthy people has been previously tested (6–8). This vaccine improves the magnitude of HIV-specific T cell responses (6, 7), although it does also tilt the balance between activation and regulation of T cell specific responses toward regulation (9), somehow explaining the viral rebound after removal of cART in patients that has received the vaccine. However, the effects of vaccination in other immune cells have not been studied. Considering that monocytes play an important role in chronic inflammation characteristic of HIV-1-infected subjects (11), we studied the phenotype and cytokine production of monocytes in HIV-1-infected patients after vaccination with MVA-B and we compared them with monocytes from the same patients before vaccination.

**Figure 2:**
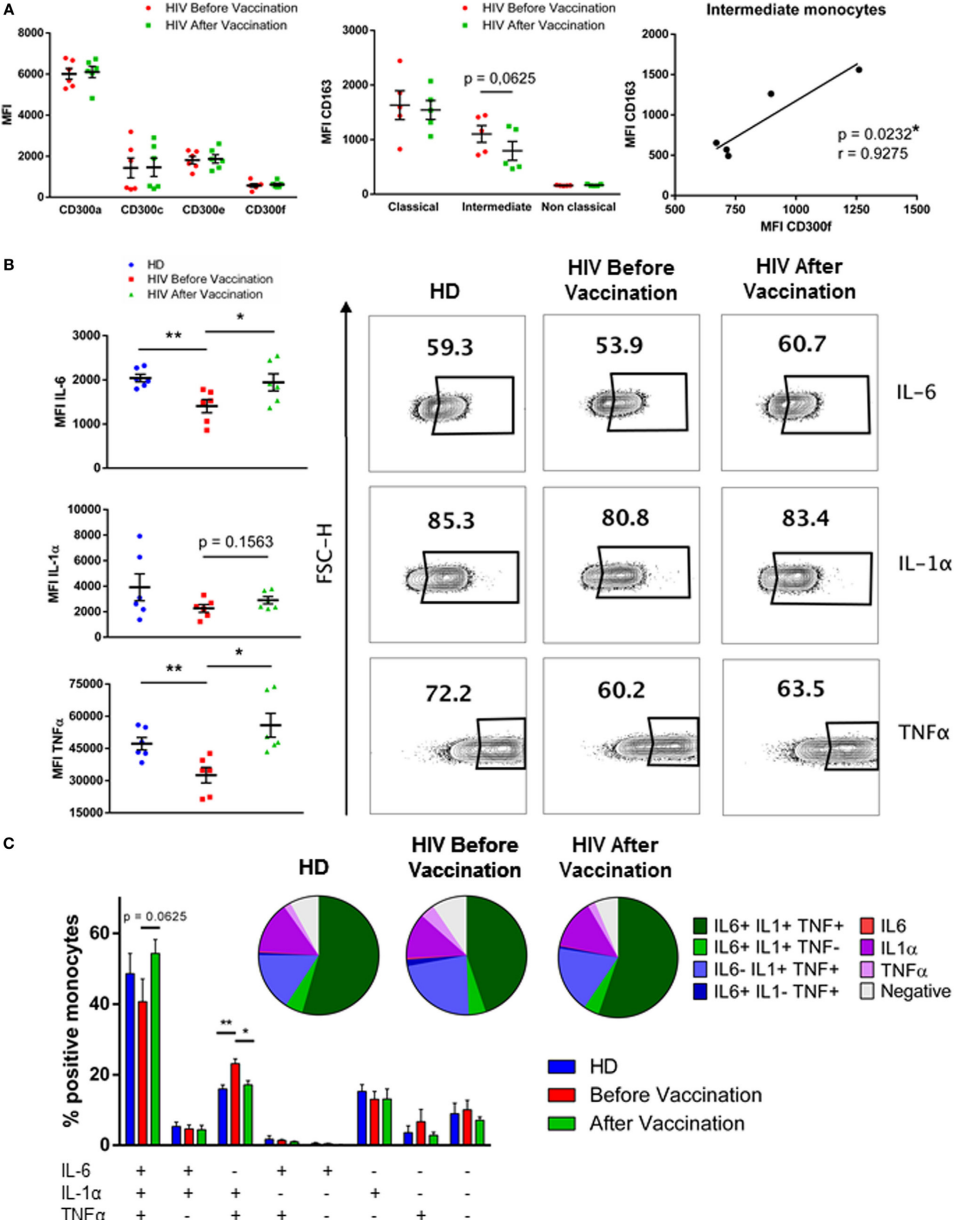
Phenotypical analysis and cytokine production of monocytes from HIV-1-infected patients after vaccination with MVA-B vaccine. **(A)** Dot plot graph (left panel) displaying the median fluorescence intensity (MFI) of CD300a, CD300c, CD300e, and CD300f receptors expression on monocytes from HIV-1-infected patients before (HIV before vaccination) and after (HIV after vaccination) vaccination. Each dot corresponds to an individual and the mean with SEM is shown. Dot plot graph (middle panel) representing the MFI of CD163 receptor expression on classical, intermediate, and non-classical monocytes from HIV-1-infected individuals before and after vaccination. Each dot corresponds to an individual and the mean with SEM is shown. The correlation between the MFI of CD163 receptor and the MFI of CD300f on intermediate monocytes of HIV-1-infected patients is represented (right panel); the linear regression is shown. **(B)** Dot plot graphs showing the MFI of positive monocytes for each cytokine; the mean with SEM is represented (left). Contour plots representing the percentage of positive monocytes for each cytokine after stimulation with lipopolysaccharide. Data from a representative healthy donor (HD) and an HIV-1-infected patient before and after vaccination are shown (right). **(C)** Boolean gate analysis representing the percentages of monocytes producing IL-6, IL-1α, and TNFα, in HD and HIV-1-infected patients before and after vaccination. Bar graphs showing the mean with SEM and pie charts are represented. *p < 0.05, **p < 0.01.

First, the expression of CD163 and CD300 surface receptors was determined in HIV-1-infected patients before and after the vaccination with MVA-B. The percentages of monocyte subpopulations in vaccinated HIV-1-infected individuals were very similar to the percentages found before the vaccination (Figure S2 in Supplementary Material). The expression of CD300 molecules was determined and we observed that the expression pattern in monocytes of HIV-1-infected patients before and after vaccination was almost identical (Figure 2A, left panel). CD163 expression on monocytes was not significantly different when compared before and after vaccination. However, on intermediate monocytes (HIV before vaccination MFI = 1,103 ± 153.4 vs HIV after vaccination MFI = 793.6 ± 173.8), CD163 tended, although not statistically significant, to be down-regulated in patients after vaccination, while in classical and non-classical monocytes CD163 expression was very similar before and after vaccination (Figure 2A, middle panel). Lastly, we analyzed the correlation between the expression of CD300 receptors and CD163 receptor, and no significant values were observed in any case, except for a positive correlation between the levels of CD300f and CD163 (*p* < 0.05, *r* = 0.9275) on intermediate monocytes, as it was found before vaccination (Figure 2A, right panel).

Afterward, PBMCs from HD and HIV-1-infected patients, before and after vaccination, were stimulated with 1 ng/ml of LPS for 5 h, followed by ICS in order to study IL-6, IL-1α, and TNFα production in monocytes. These were gated according to their forward and side scatter properties, and they were defined as CD14++ HLA−DR+. In our hands, monocyte subpopulations were not distinguished due to the down-regulation of CD16 receptor after LPS stimulation (data not shown). Positive cells for each cytokine were determined based on non-stimulated cells. First, we checked the level of cytokine production by the stimulated cells by MFI of cytokine staining, a value known to be correlated with the amount of cytokine produced by cells (21). We observed that monocytes from HIV-1-infected subjects produced less IL-6 and TNFα than monocytes from HD in response to LPS. Interestingly, monocytes of vaccinated HIV-1-infected patients produced higher levels of IL-6, IL-1α, and TNFα in response to LPS after vaccination. Although IL-6 levels in vaccinated patients remained lower than in HD, TNFα production in vaccinated subjects reached the same levels as those from HD (Figure 2B). Moreover, analysis showed that the percentage of triple positive (IL-6+IL-1α+TNFα+) monocytes in response to LPS was higher in vaccinated HIV-1-infected subjects compared with the percentage of triple positive monocytes from the same patients before vaccination. On the other hand, the percentage of only double positive (IL-6-IL-1α+TNFα+) monocytes was higher in patients before the vaccination. These results indicate that monocytes of HIV-1-infected subjects were more polyfunctional in response to LPS stimulation after vaccination than before vaccination. As expected, although differences were not significant, probably due to the small sample, it was observed a higher percentage of non-cytokine (IL-6−IL-1α−TNFα−) producing monocytes from patients before vaccination than in monocytes after vaccination and from HD (HD = 7.63% vs HIV no vaccinated = 9.23% vs HIV vaccinated = 6.45%) (Figure 2C). In conclusion, monocyte cytokine production in response to LPS in HIV-1-infected patients was higher after vaccination and resembled that observed in HD.

### Relationship between CD300 Receptors Expression and Cytokine Production by Monocytes of HIV-1-Infected Patients Before and After Vaccination

**Figure 3:**
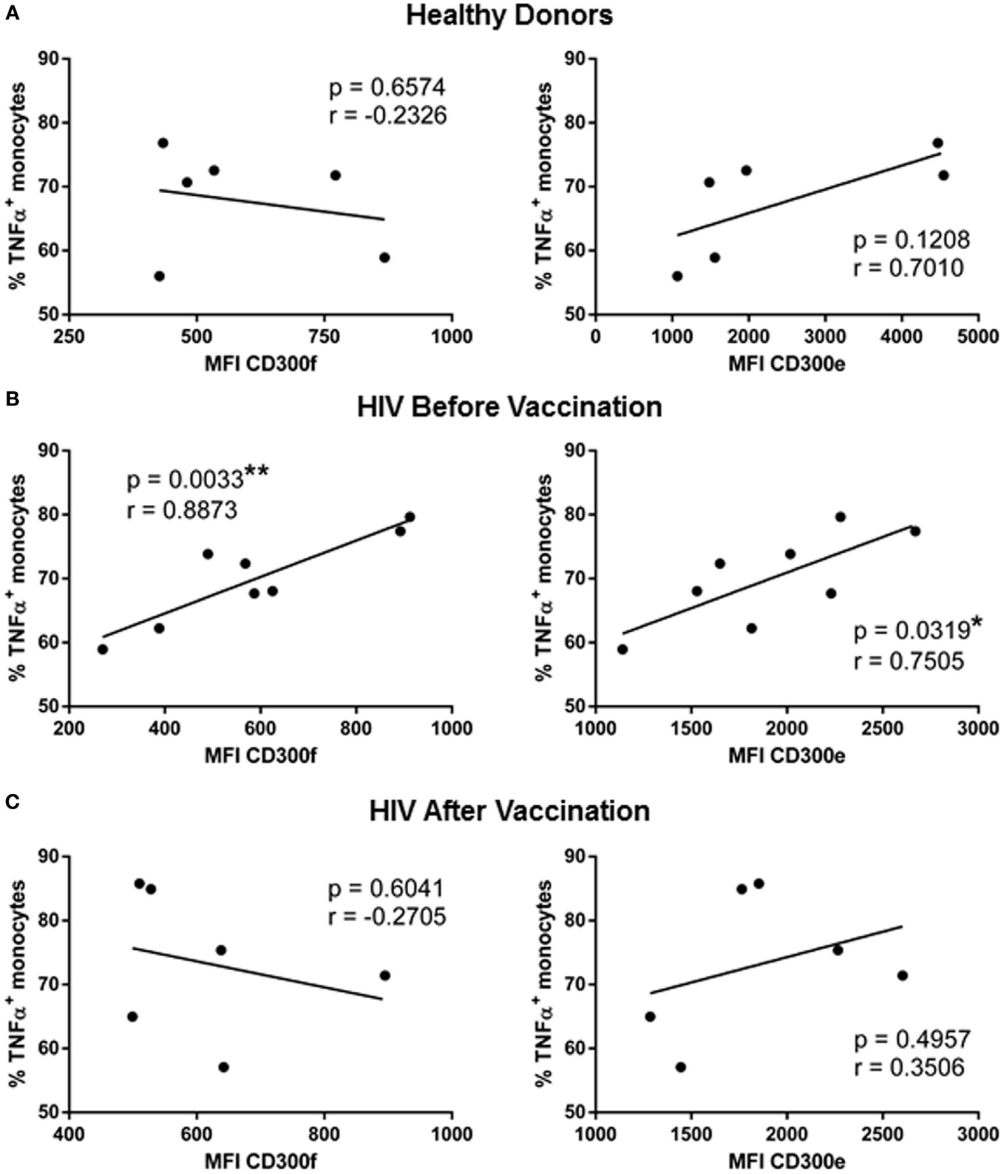
Correlation analysis of TNFα production with the expression of CD300 receptors in human immunodeficiency virus (HIV)-1-infected patients before and after vaccination with MVA-B. Representation of the correlation between the percentage of TNFα positive monocytes and the median fluorescence intensity of CD300f and CD300e receptors expression, in healthy donors **(A)** and HIV-1-infected patients before **(B)** and after **(C)** vaccination with MVA-B; the linear regression is shown in each graph. *p < 0.05, **p < 0.01.

The last step of the work was to investigate if the expression levels of CD300 molecules could have a correlation with the increased functionality found after the MVA-B vaccination in monocytes of HIV-1-infected individuals. We performed correlation analysis between CD300 receptors expression and cytokine production in response to LPS. The expression of CD300 molecules was not correlated with the percentage of IL-6+ monocytes in any case. In contrast, the expression of CD300e and CD300f correlated with IL-1α and TNFα production. The correlation with IL-1α production was only observed in monocytes from HD (data not shown); however, the expression of CD300e (*p* < 0.05, *r* = 0.7505) and CD300f (*p* < 0.01, *r* = 0.8873) was positively correlated with TNFα production in monocytes of HIV-1-infected patients before vaccination (Figure 3B). The percentages of TNFα+ monocytes of HD and vaccinated patients were not correlated with the MFI of CD300e and CD300f (Figures 3A,C). In fact, as it can be observed in the graphical representation (Figure 3), monocytes from HIV-1-infected patients are more similar to those from HD than to the monocytes from the same patients before vaccination. Taking altogether, we could propose that the monocyte phenotype and functional pattern in response to LPS stimulation of HIV-1-infected patients after vaccination with MVA-B are more similar to those found in monocytes from HD than from monocytes from HIV-1-infected subjects before vaccination.

## Discussion

Monocytes have been described as one of the cell types involved in the chronic inflammation characteristic of cART-treated HIV-1-infected people, which is currently the cause of death of the majority of HIV-1-patients (11). High numbers of circulating intermediate and non-classical monocytes have been associated with inflammation and immune activation during HIV infection (10). Furthermore, inflammatory mediators (e.g., IL-6) secreted by monocytes predict serious non-AIDS events in virologically suppressed HIV-infected subjects (11). Three main mechanisms have been proposed to explain the monocyte activation and consequently, the inflammation found in cART-treated HIV-infected patients: the microbial translocation, which augments LPS levels in plasma, the residual HIV viremia, and coinfection with human cytomegalovirus or some herpesviruses (11).

Since the CD300 family of receptors are able to modulate monocytes function (20, 22–24), our first objective was to investigate the CD300 receptors expression in monocytes from cART-treated chronically HIV-1-infected patients. Our results revealed that the expression pattern of CD300 molecules in monocytes from HD and in monocytes from HIV-1-infected people were not significantly different. However, we observed that the expression of CD300c tended, although not statistically significant, to be down-regulated in monocytes from HIV-1-infected patients, in comparison with monocytes from HD. This could be explained in part with the increase of the percentage of non-classical monocytes in HIV-1-infected patients, which express lower levels of CD300c than classical monocytes (Figure S3 in Supplementary Material) (20). It is important to keep in mind that many immunological abnormalities observed during the course of HIV infection can be reversed by cART, and therefore it is possible that the expression of CD300 molecules is altered in non-cART-treated patients with detectable viremia. More studies with blood samples from viremic patients are needed to obtain a more complete picture on the expression of the CD300 molecules during HIV infection. We did found a significant correlation between the expression of the activating receptor CD300e and the inhibitory receptor CD300f in monocytes with CD4+ T cell count in patients whose viremia is controlled by undergoing cART. These results may suggest that the levels of expression of CD300e and CD300f on monocytes could potentially be used as biomarkers of disease progression in combination with the well know predictive value of CD4+ T cell count (25, 26). Prospective studies with larger cohorts will confirm the predictive value of CD300e and CD300f expression on monocytes from HIV-infected patients.

We have not seen a significant increase in the expression of CD163 on monocytes from HIV-infected patients compared with monocytes from HD. Somehow, our results are different from those reported by others (13). We believe that this discrepancy is due to the low number of patients we have studied, since it is possible to observe a tendency, although not statistically significant, to increase CD163 cell surface expression on monocytes from HIV-infected individuals. Interestingly, there was a positive correlation between the expression of CD300f and CD163 in intermediate monocytes, a subset with a significant role in inflammation (27). The positive correlation between the expression of CD300f and CD163 was maintained after vaccination. These results also suggest that the expression of CD300f, along with other markers, could be used as a biomarker of inflammation in HIV-infected patients. Human and mouse CD300f is commonly considered an inhibitory receptor because of the presence of ITIMs motifs in its intracellular tail (14). Several publications have shown its inhibitory role on monocyte cell lines (28–30). However, it has also been demonstrated that CD300f is able to deliver activating signals through motifs reported to bind the p85α regulatory subunit of PI3K (YxxM) (31–33). *In vivo* models in mice have shown that CD300f both inhibits and promotes the development of autoimmune diseases and allergic and inflammatory responses (34–39). This dual role of CD300f somehow may depend, not only on the cell type this intriguing receptor is expressed, but also on its described association with other receptors and adaptor proteins (33, 38, 40, 41). It would be of great interest to determine the signaling pathways of CD300f on monocytes during HIV infection, and determine if this receptor has different roles in monocytes from HD and HIV-1-infected patients.

Several therapeutic vaccines have been tested with the objective of controlling viral replication and to avoid viral rebound after treatment interruption in chronically HIV-1-infected patients (42, 43). MVA-B is an immunogenic vaccine which induces a T cell response in HIV-1-infected patients (7, 8). As expected, we did not observe any significant differences in the expression of CD300 molecules in monocytes of HIV-1-infected patients before and after vaccination. The most intriguing finding of this study was that the response of monocytes to LPS stimulation from patients after vaccination was different from the response before the vaccination, and at the same time similar to the response of monocytes from HD. Monocytes from non-vaccinated HIV-1-infected patients produced less cytokines in response to LPS than HD. This is in agreement with previous findings showing that HIV impairs TNFα production by human macrophages in response to Toll-like receptor 4 stimulation (44). Furthermore, this lower production of cytokines could also be due to the fact that monocytes when are chronically stimulated *in vivo* during chronic HIV infection become refractory to further stimulation with LPS *in vitro* (45), and it has been published that ART-treated infected patients exhibit higher levels of LPS in plasma than HD (46).

Vaccination with MVA-B induced higher levels of IL-6, IL-1α, and TNFα by monocytes in response to LPS. In fact, monocytes of vaccinated subjects exhibited a functional pattern more similar to the one of HD than to non-vaccinated HIV-1-infected patients. Furthermore, when we investigated if the expression of CD300 receptors might be correlated with the cytokine production levels, we also observed that the results were comparable between HD and HIV-1-infected patients after vaccination, and not between patients before and after vaccination. For example, the expression of CD300e and CD300f was positively correlated with TNFα levels in monocytes of HIV-1-infected subjects before vaccination, but not after vaccination or in monocytes of HD. We do not know the causes of this increase in the production of pro-inflammatory cytokines by monocytes in response to LPS after vaccination and if our results have some role in the lack of efficacy of the MVA-B vaccine as shown by a viral rebound after treatment interruption. It is possible that tipping the balance between activation and regulation toward regulation of the response of HIV-specific CD8+ T cells is not the only factor responsible for the lack of efficacy of the MVA-B vaccine. On the one hand, and considering our results showing lower CD163 expression on monocytes after vaccination, it seems that the administration of MVA-B vaccines may favor a less inflammatory environment. However, on the other hand, monocytes after vaccination have the potential to produce higher levels of pro-inflammatory cytokines and therefore could help to explain the lack of efficacy of the vaccine due to higher inflammation (10, 47–49). Also, it is important to remember that these patients have received disulfiram along with the MVA-B vaccine. Although the effect of disulfiram in monocytes of HIV-1-infected patients is unknown, several publications suggest that this drug have a role in decreasing the production of inflammatory mediators by monocytes. For example, it has been described that this compound diminishes the number of inflammatory cells and TNFα levels in the aqueous humor, in rats with endotoxin-induced uveitis (50). Furthermore, diethyldithiocarbamate, the active compound produced *in vivo* from disulfiram, impairs the release of oxygen metabolites and prostaglandins of human monocytes, two major pathways related to inflammatory processes (51). Undoubtedly, further research is required to delineate the role of monocytes in the efficacy of therapeutic vaccines.

In conclusion, our results have shown that vaccination with MVA-B, in addition to induce a specific T cell response, has also an effect on monocytes phenotype and their ability to produce cytokines after stimulation with LPS. We acknowledge that the number of patients included in this study is low and that it is very possible that a higher number of patients will provide more robust results. Clearly, more studies would be required to determine if the MVA-B mediated effect on monocytes favors the efficacy of the vaccine, or by the contrary is counterproductive. However, we believe that the results obtained with this work may form the basis of future studies to determine the functionality and phenotype of monocytes from patients enrolled in clinical trials testing therapeutic vaccines.

## Ethics Statement

This study was carried out in accordance with the recommendations of Ethical Committee of Hospital Clìnic, Barcelona, Hospital Germans Trias i Pujol, Badalona, and Hospital Gregorio Marañón, Madrid, Spain with written informed consent from all subjects. All subjects gave written informed consent in accordance with the Declaration of Helsinki. The protocol was approved by the Ethics Committee of Hospital Clínic, Barcelona, Hospital Germans Trias i Pujol, Badalona, and Hospital Gregorio Marañón, Madrid, Spain.

## Author Contributions

JV designed the study, designed and performed experiments, analyzed, and interpreted the data, designed the figures, and wrote the manuscript. OZ performed experiments and interpreted the data. IT designed the figures. MP participated in the design of the study and interpreted the data. AG participated in analysis and interpretation of the data. LL recruited and followed the patients and was responsible of vaccinations and clinical monitoring. JP interpreted the data. FG recruited and followed the patients and was responsible of vaccinations and clinical monitoring. FB conceived and designed the study, interpreted the data, and wrote the manuscript. All the authors critically reviewed, edited, and approved the final manuscript.

## Conflict of Interest Statement

The authors declare that the research was conducted in the absence of any commercial or financial relationships that could be construed as a potential conflict of interest.

## Members of the RISVAC-03 Study Group

*Irsicaixa AIDS Research Institute-HIVACAT, Hospital Germans Trias i Pujol, Badalona, Spain*: Beatriz Mothe, Patricia Cobarsi, Miriam Rosàs, M. Carmen Puertas, Jorge Carrillo, Juliá Blanco, Javier Martínez-Picado, Bonaventura Clotet, Christian Brander.

*Hospital Clinic-HIVACAT, IDIBAPS, University of Barcelona, Spain*: Nuria Climent, Montserrat Plana, Carmen Alvarez, Sonsoles Sánchez, Agathe León, Judit Pich, Joan Albert Arnaiz, Lorna Leal, Berta Torres, Constanza Lucero, Alberto C. Guardo, Jose M. Gatell, Felipe García.

*Hospital Gregorio Marañón, Madrid, Spain*: José Luis Jiménez, María Angeles Muñoz-Fernández, Juan Carlos López Bernaldo de Quirós.

*Centro Nacional de Biotecnología, CSIC, Madrid, Spain*: Mariano Esteban, Carmen Elena Gómez, Beatriz Perdiguero, Juan García-Arriaza, Victoria Cepeda, Carlos Oscar Sánchez-Sorzano.

*Instituto de Salud Carlos III, Madrid, Spain*: Nuria Gonzalez, José Alcamí, Laura Jiménez.

*Instituto de Investigación Sanitaria—Fundación Jiménez Díaz, Madrid, Spain*: José M Benito, Norma Rallón.

*Hospital Reina Sofía, Córdoba, Spain*: José Peña.
