# Supplementary figures and images for: Monocytes Phenotype and Cytokine Production in Human Immunodeficiency Virus-1 Infected Patients Receiving a Modified Vaccinia Ankara-Based HIV-1 Vaccine: Relationship to CD300 Molecules Expression
Source: Front Immunol. 2017 Jul 21;8:836. doi: 10.3389/fimmu.2017.00836 (PMC5520290; doi:10.3389/fimmu.2017.00836)

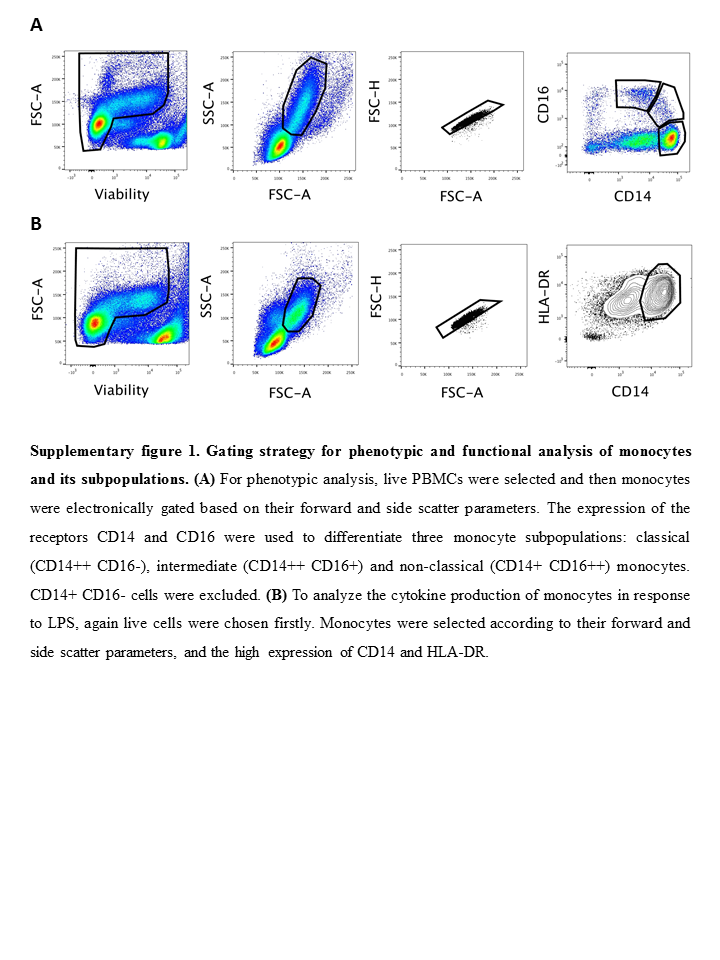

Supplement: Supplementary file 1 [file Image_1.TIF]

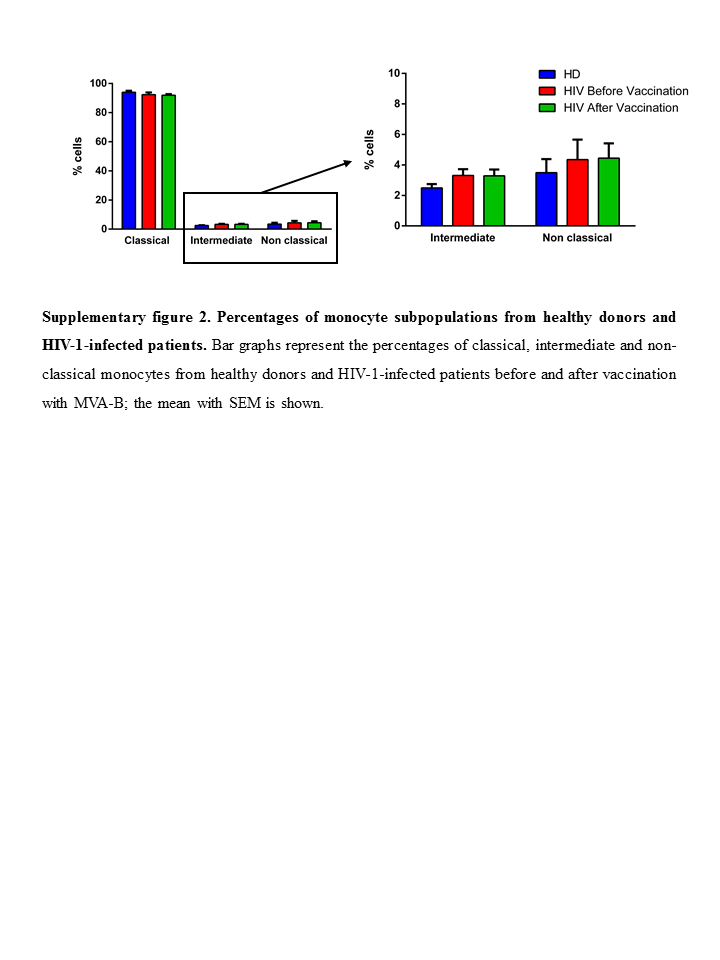

Supplement: Supplementary file 2 [file Image_2.TIF]

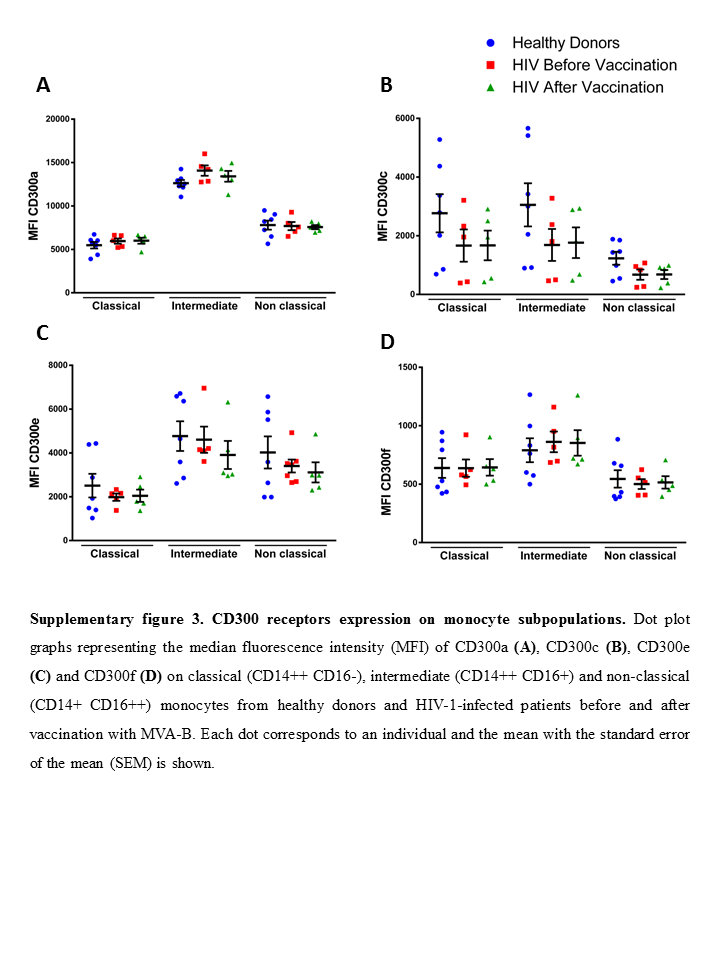

Supplement: Supplementary file 3 [file Image_3.TIF]
